# Probiotic-Derived Polyphosphate Accelerates Intestinal Epithelia Wound Healing through Inducing Platelet-Derived Mediators

**DOI:** 10.1155/2021/5582943

**Published:** 2021-03-29

**Authors:** Shotaro Isozaki, Hiroaki Konishi, Mikihiro Fujiya, Hiroki Tanaka, Yuki Murakami, Shin Kashima, Katsuyoshi Ando, Nobuhiro Ueno, Kentaro Moriichi, Toshikatsu Okumura

**Affiliations:** ^1^Division of Metabolism and Biosystemic Science, Gastroenterology, And Hematology/Oncology, Department of Medicine, Asahikawa Medical University, Asahikawa 078-8510, Japan; ^2^Department of Gastroenterology and Advanced Medical Sciences, Asahikawa Medical University, Asahikawa 078-8510, Japan; ^3^Division of Tumor Pathology, Department of Pathology, Asahikawa Medical University, Asahikawa 078-8510, Japan

## Abstract

Inflammatory bowel disease (IBD), such as ulcerative colitis (UC) and Crohn's disease (CD), is an intractable intestinal inflammation associated with the disruption of the intestinal mucosa. We previously demonstrated that *Lactobacillus brevis*-derived long-chain polyphosphate (poly P) improved the intestinal barrier function by the upregulation of cell adhesion and relieved intestinal inflammation, thereby exerting a curing effect on colitis *in vitro*, *in vivo*, and in an investigator-initiated clinical study of UC. However, how poly P improves mucosal defects induced by intestinal inflammation has not been elucidated. In this study, we detected the accumulation of platelets in inflamed tissues induced by poly P in a dextran sulfate sodium- (DSS-) induced colitis mouse model. A light transmission aggregometry analysis and scanning electron microscopy showed that poly P promoted the platelet aggregation. An SRB assay and ki-67 staining showed that the supernatant of poly P-treated platelet-rich plasma (PRP) increased intestinal epithelial cell growth. A wound healing assay showed that the supernatant of poly P-treated PRP, but not poly P itself, accelerated wound healing. A Western blotting analysis indicated that mitogen-activated protein kinase activation was induced by the supernatant of poly P-treated human PRP in the epithelial cells and its wound healing effect was significantly decreased by the inhibition of ERK signaling. These data suggested that platelet-derived mediators induced by poly P improved intestinal inflammation through the promotion of epithelial cell growth by the activation of the ERK signaling pathway. The mechanism is a novel host-microbe interaction through mammalian platelet-derived mediators induced by bacterial molecules.

## 1. Introduction

Various bacteria live in the host gastrointestinal tract and build a symbiotic relationship with their mammalian hosts. It has been shown that the disruption of the intestinal microflora results in harm to the host, such as the abnormal activation of immune systems and consequently the development of various diseases, including gastrointestinal inflammations and tumors, liver dysfunction, atopic dermatitis, and brain and neurological disorders [1–3].

Previous studies have suggested that the improvement of the intestinal condition by the administration of beneficial bacteria, such as *Lactobacilli* and *Bifidobacterium*, helped resolve these diseases in *in vitro* and *in vivo* animal experimental models [4–6]. Other studies, including our own, have shown that some probiotic bacteria released bioactive mediators that contributed to the maintenance of intestinal homeostasis and the improvement of inflammation. Yan et al. identified p40 and p75 from the culture supernatant of *Lactobacillus GG* and showed how their anti-inflammatory effects mediated the epithelial EGFR-Akt signaling pathway [7]. Likewise, we identified competence and sporulation factor (CSF) from *Bacillus subtilis* and showed that CSF was incorporated into epithelial cells through OCTN2, thus activating the cell survival signaling, including the Akt and p38 mitogen-activated protein kinase (MAPK) pathways, resulting in an increase in the epithelial barrier function [8]. Kelly et al. found that microbiota-derived short-chain fatty acids stabilize the hypoxia-inducible factor (HIF) and strengthen the gut epithelial barrier function [9].

We also identified long-chain polyphosphate (poly P) as a beneficial substance in the culture supernatant of *L. brevis* SBC8803 that supports the intestinal barrier function of the mammalian host through the integrin *β*1-p38 MAPK signaling pathway [10]. Furthermore, poly P upregulated the intestinal barrier function and downregulated the macrophage activation in a murine colitis model and dramatically improved the symptoms and endoscopic findings of patients with refractory ulcerative colitis (UC) in an investigator-initiated clinical trial [11, 12]. Taken together, these previous findings support the notion of host-microbe interaction mediated by probiotic-derived molecules through the modulation of epithelial and immune cells.

Excess intestinal inflammation, such as inflammatory bowel disease, has been known to cause mucosal defects, including epithelial erosion and ulcers, which lead to the translocation of bacteria and/or harmful components, thereby exacerbating the intestinal inflammation [1, 13, 14]. Because an investigator-initiated clinical trial showed that poly P frequently induces endoscopic mucosal healing in refractory UC patients who exhibited multiple erosions and ulcers, we speculated that poly P was effective for treating mucosal defects as well as improving the intestinal barrier function [12]. However, the mechanism underlying the promotion of wound healing by poly P has not been elucidated. In the present study, we detected, for the first time, the aggregation of platelets, which is known to promote wound closure [15], at the surface of the mucosa in a DSS-induced colitis model after treatment with poly P. We therefore focused on the wound healing effect of poly P through the crossinteraction between poly P and host platelet-derived small-molecule mediators in *in vitro* wound healing models and a mouse enteritis model.

## 2. Materials and Methods

### 2.1. Poly P

A mixture of 200 mM phosphoenolpyruvic acid (2 M Tris-HCl, pH 9.4), 30 mM phosphate buffer (pH 6.0), 30 mM adenosine triphosphate (1 M Tris-HCl, pH 8.0), 30 mM MgCl_2_, 600 mM acetic acid buffer (pH 6.0), 0.25 mL poly P kinase, and 100 U/mL pyruvate kinase was incubated at 37°C for 16–18 h. To purify the poly P, the mixture was incubated with the same volume of 2 M acetic acid buffer at 4°C for 2 h, and then, this was centrifuged at 3000 rpm for 5 minutes. The precipitate was dissolved in distilled water and incubated with the same volume of 2 M acetic acid buffer at 4°C for 2 h. After another round of centrifugation, the precipitate was dissolved in distilled water and the low-molecular weight components, including adenosine triphosphate and short-chain poly P, were removed from the solution by dialysis using a tube equipped with a 3 kDa molecular weight cutoff (MWCO) membrane (Thermo Fisher Scientific Ltd., Waltham, MA, USA). Poly P was dissolved in distilled water at 20 mg/mL and adjusted to pH 10. CaCl_2_ was added to the poly P solution in a molecular ratio (Ca : P = 1 : 2) using a peristaltic pump. Insoluble poly P was collected using an Amicon Ultra 3K filter device (Merck KGaA, Darmstadt, Germany) at 3000 rpm, and ethanol was added and centrifuged at 3000 rpm. The poly P was dried using a vacuum drying pump.

### 2.2. Short-Chain Polyphosphate

Sodium hexametaphosphate was purchased from FUJIFILM Wako Pure Chemical Corporation, Osaka, Japan. Sodium hexametaphosphate was dissolved in distilled water at 20 mg/mL and adjusted to pH 10. CaCl_2_ was added to the poly P solution in a molecular ratio (Ca : P = 1 : 2) using a peristaltic pump. Insoluble short-chain polyphosphate was collected using an Amicon Ultra 3K filter device (Merck KGaA) at 3000 rpm, and ethanol was added and centrifuged at 3000 rpm. The short-chain polyphosphate was dried using a vacuum drying pump.

### 2.3. Animals

The studies were approved by the Institutional Animal Care and Use Committee of the Asahikawa Medical University (permit number 19027-2). BALB/c mice and Wistar Rattus were purchased from Charles River Laboratories Japan Inc. (Yokohama, Japan).

### 2.4. The Induction and Assessment of Colitis

BALB/c mice were administered with 2.5% (wt/vol) dextran sulfate sodium (DSS) (molecular weight, 36–50 kDa) in the drinking water for 5 days and then switched to distilled water after administration. In the test group (*n* = 8), 5 *μ*g of *L. brevis*-derived poly P dissolved in 100 *μ*L of phosphate-buffered saline (PBS) was perorally administered once a day throughout the experimental period. In the control group (*n* = 8), 100 *μ*L of PBS was perorally administered once a day throughout the experimental period. The mice were sacrificed on the 7th day, and the entire colon was removed from the cecum to the anus. The colon length was then measured as a marker of inflammation. A few pieces of colon mucosa were collected to assess the mRNA expression using real-time reverse transcription polymerase chain reaction (RT-PCR), and the other pieces were fixed in 10% buffered formalin, sectioned at 4 *μ*m, and used for hematoxylin and eosin staining followed by a light microscopy analysis.

### 2.5. Histopathological Inflammation Score

The histological changes were assessed according to Berg's score [16]. The grade of intestinal inflammation was assessed at three representative parts of the colon in each mouse because the intestinal lesions were multifocal and the severity of the intestinal lesions varied. The score was determined as follows: grade 0, no change from normal tissue; grade 1, one or a few multifocal mononuclear cells infiltrating the lamina propria accompanied by minimal epithelial hyperplasia and slight to no depletion of mucus from goblet cells; grade 2, the lesions tend to involve more of the intestine than grade 1 lesions or are more frequent, typical changes include mild inflammatory cell infiltrates in the lamina propria primarily composed of mononuclear cells with a few neutrophils, small epithelial erosions are occasionally present, and inflammation rarely involves the submucosa; grade 3, lesions involve a large area of the mucosa or are more frequent than grade 2 lesions, inflammation is moderate and often involves the submucosa but is rarely transmural, inflammatory cells are a mixture of mononuclear cells as well as neutrophils, and crypt abscesses and ulcers are occasionally observed; and grade 4, such lesions usually involve most of the intestinal section and are more severe than grade 3 lesions, with severe inflammation, including mononuclear cells and neutrophils, that is, sometimes transmural, while crypt abscesses and ulcers are present.

### 2.6. Real-Time RT-PCR

Total RNA was extracted from the colorectal tissue of mice using TRIzol (Invitrogen) and purified with an RNeasy Mini Kit (QIAGEN, Tokyo, Japan) according to the manufacturer's instructions. The quality and quantity of total RNA were verified by spectrophotometry. Single-stranded complementary DNA (cDNA) was synthesized using a High-Capacity cDNA Reverse Transcription Kit (Applied Biosystems, Foster City, CA, USA). The gene expression was measured with real-time PCR using specific primers of TNF-*α*, IFN-*γ*, IL-6, and IL-1*β* (Thermo Fisher Scientific). The average mRNA expression was normalized to the 18S rRNA expression.

### 2.7. Immunohistochemistry

Formalin-fixed, paraffin-embedded tissue samples were prepared in accordance with a standard protocol. CD61 was stained as a cell surface marker of platelets. For the immunohistochemical analysis, paraffin sections were sequentially treated before application of the primary antibody in the following way: deparaffinization, rehydration, quenching of endogenous peroxidase, and antigen retrieval. Sections were then incubated with primary antibody for anti-CD61 (Cell Signaling Technology, Danvers, MA, USA), followed by detection with horseradish peroxidase-conjugated secondary antibody for mice or rabbits (Vector Labs, Burlingame, CA, USA). The CD61-positive area in the colon was quantified. Seven representative images of the colon from each mouse were taken under ×40 magnification. After obtaining each CD61-positive area using the ImageJ software program [17], the ratio of each area to the average area in control mice was calculated.

### 2.8. Platelet-Rich Plasma (PRP) Preparation

Blood samples were collected from the postcaval vein in rats or peripheral vein in healthy human volunteers. The studies were approved by the ethics committee of Asahikawa Medical University (number, 19015-2), and informed consent of each volunteer was obtained. Blood samples were drawn into sample tubes containing buffered 3.2% sodium citrate and centrifuged at 800 g for 10 min, and PRP was collected from the supernatant.

### 2.9. Preparation of the Supernatant of Poly P-Treated PRP

Poly P was diluted in Hanks' Balanced Salt Solution (HBBS) in 10 mg/mL and added to the PRP (final concentration of poly P: 1 mg/mL), after which it was rotated for 20 min at room temperature. The platelet aggregates were pelleted by centrifugation for 10 min in a microcentrifuge at 2400 g, and poly P-treated plasma was obtained for use in the enzyme-linked immunosorbent assay (ELISA). To eliminate clotting factors, plasma was separated by a 100 kDa molecular cutoff (MWCO) column (GE Healthcare, Chicago, IL, USA).

### 2.10. Light Transmission Aggregometry

Aggregation responses to HBSS, poly P (0.5 mg/mL), and ADP (40 *μ*M) (FUJIFILM Wako Pure Chemical) were measured (*n* = 5) using an EnSpire (PerkinElmer Japan, Kanagawa, Japan).

### 2.11. Scanning Electron Microscopy

Scanning electron microscopy was used to study the morphology of the platelet aggregates formed in response to poly P (0.1 mg/mL) or ADP (40 *μ*M). Following incubation for 20 min, platelet aggregates were pelleted by centrifugation for 10 min in a microcentrifuge at 2400 g. Plasma was removed and fixed in 4% paraformaldehyde, followed by further fixation in 1% osmium tetroxide with PBS. Specimens were examined using a Hitachi S-4100 (Hitachi High-Tech Corporation, Tokyo, Japan) at the Center for Advanced Research and Education, Asahikawa Medical University, Asahikawa, Japan.

### 2.12. Cell Culture

IEC-18 cells were grown in high-glucose Dulbecco's Modified Eagle's Medium (DMEM) (FUJIFILM Wako Pure Chemical) supplemented with 10% (vol/vol) fetal bovine serum (FBS), 2 mM L-glutamine, 50 U/mL penicillin, and 50 mg/mL streptomycin (Thermo Fisher). HCEC-1CT cells were grown in ColoUp medium (Evercyte, Wien, Austria). Both cell lines were incubated in a humidified atmosphere of 5% CO_2_.

### 2.13. A Wound Healing Assay

The cells were seeded on 12-well microplates at 10^5^ cells per well and cultured for 24 h. The scratches were created with a sterile 200 *μ*L pipette tip, and then, the supernatant of HBSS or poly P or ADP-treated PRP diluted twofold by culture medium was added to the cells. The wound areas were recorded using a digital camera system. The distance of scratches was measured using the ImageJ software program.

### 2.14. Confocal Immunofluorescence Microscopy

HCEC-1CT cells were plated on chamber slides and allowed to culture in the supernatant of HBSS or poly P-treated PRP for 6 h. Slides were then fixed for 15 min in 4% paraformaldehyde, washed extensively with PBS, permeabilized with 0.2% Triton X-100 for 30 min, and blocked in SuperBlock Blocking Buffer (PBS) (Thermo Fisher) for 1 h at room temperature. Slides were then sequentially incubated with primary antibodies Ki-67 (Cell Signaling Technology) for 24 h at 4°C, washed with PBS, and then incubated with anti-rabbit antibody conjugated with Alexa Fluor 488 (Thermo Fisher) for 1 h. The nuclei were counterstained with Hoechest33342 (Thermo Fisher) for 3 min. The cells were mounted with an antifade mounting medium, and immunofluorescence was visualized using a confocal microscope. The Ki-67-positive rate (%) was calculated based on the proportion of the Ki-67-positive area to the Hoechst-positive area, which was measured using the ImageJ software program.

### 2.15. SRB Assay

HCEC-1CT cells were seeded on 96-well microplates at 1 × 10^4^ cells/well and cultured for 24 h. The cells were fixed in 5% trichloroacetic acid (TCA) for 1 h at 4°C and washed 4 times in distilled water. The microplates were then dehydrated at room temperature, stained in 100 mL per well of 0.057% (wt/vol) SRB powder/distilled water, washed 4 times in 0.1% acetic acid, and redehydrated at room temperature. The stained cells were lysed in 10 mM Tris buffer, and the optical density (OD) was measured at 510 nm [18].

### 2.16. Western Blotting

IEC-18 cells were seeded on 12-well microplates at 1 × 10^5^ cells per well and cultured for 24 h. Cells were treated with the supernatant of HBSS or poly P-treated PRP diluted twofold with medium. After incubation for 30 min, the cells were lysed in NP-40 cell lysis buffer (Thermo Fisher) containing phosphatase inhibitor (Thermo Fisher) and a complete protease inhibitor cocktail (Roche Molecular Biochemicals, Indianapolis, IN, USA). Protein (20 *μ*g) in each sample was resolved using sodium dodecyl sulfate-polyacrylamide gel electrophoresis (SDS-PAGE) (12%) and immediately transferred to a nitrocellulose membrane using transfer buffer (Bio-Rad, Hercules, CA, USA). The nitrocellulose membranes were incubated in SuperBlock Blocking Buffer (TBS) (Thermo Fisher) for 1 h at room temperature to block any nonspecific binding. The blots were incubated overnight at 4°C with anti-phospho-AKT antibody (Cell Signaling), anti-phospho-ERK antibody (Cell Signaling), antiphospho-p38 antibody (Cell Signaling), anti-phospho-JNK antibody (Cell Signaling), anti-phospho-Raf antibody (Cell Signaling), and anti-phospho-MEK antibody (Cell Signaling) as the primary antibodies.

The next day, the blots were quickly rinsed in distilled water 5 times and then washed once for 5 min with T-TBS at room temperature, incubated for 60 min in species-appropriate horseradish peroxidase-conjugated secondary antibodies (R&D Systems, Minneapolis, MN,USA) in T-TBS, quickly rinsed in distilled water 5 times, and then washed once for 5 min with T-TBS [19]. The blots were developed using the SuperSignal West Pico Enhanced Chemiluminescence System (Thermo Fisher) according to the manufacturer's protocols. The average protein expression was normalized to the actin expression (BD Transduction Laboratories, Lexington, KY, USA).

### 2.17. An ELISA

The D-dimer levels in plasma were determined using the Mouse Fibrin Degradation Product D-Dimer (Competitive EIA) ELISA Kit (LifeSpan BioSciences Inc., Seattle, WA, USA) according to the manufacturer's instructions.

The contents of growth factors in plasma were determined using the PDGF-BB, EGF, VEGF, and TGF-*β* Quantikine ELISA kits (Research and Diagnostic Systems Inc., Minneapolis, MN, USA) according to the manufacturer's instructions.

### 2.18. Inhibitors

The ERK1/2 inhibitor FR180204 (Sigma-Aldrich Co., LLC, St. Louis, MO, USA) was used at a terminal concentration of 1 *μ*M. The p38 inhibitor SB203580 (Sigma-Aldrich Co., LLC) was used at a terminal concentration of 1 *μ*M. The PI3K inhibitor LY294002 (Sigma-Aldrich Co., LLC) was used at a terminal concentration of 20 *μ*M. The JNK inhibitor SP600125 (Sigma-Aldrich Co., LLC) was used at a terminal concentration of 20 *μ*M.

### 2.19. Statistical Analyses

The assay data were analyzed using Student's unpaired *t*-test for two-group comparisons, a one-way analysis of variance (ANOVA) followed by Fisher's protected least post hoc test for three-group comparisons, and an ANOVA followed by the Tukey-Kramer method post hoc test for four-group comparisons. The growth factor ELISA data were analyzed using a paired *t*-test. The results of an SRB assay were analyzed using a two-factor repeated measure ANOVA. Wound healing assays using MAPK inhibitors were analyzed using a two-factor factorial ANOVA. *P* < 0.05 was considered to indicate a statistically significant difference.

## 3. Results

### 3.1. Poly P Improved the Intestinal Injuries of Mice Treated with DSS and Enhanced the Accumulation of Platelets in Intestinal Epithelia

**Figure 1:**
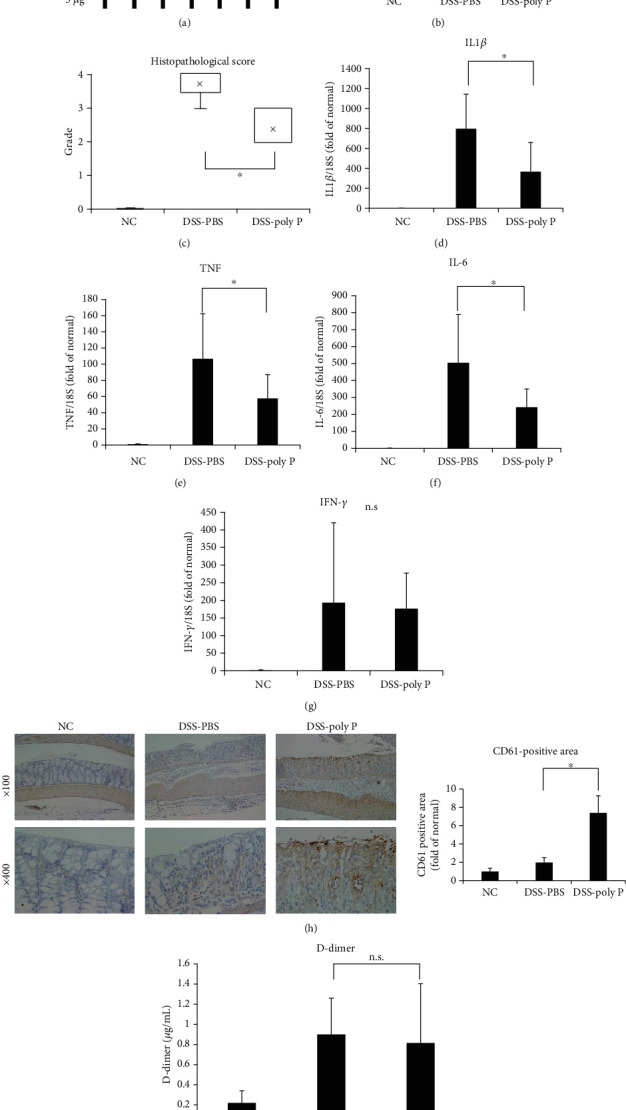
Poly P improved intestinal inflammation and enhanced the accumulation of platelets at the intestinal epithelia in a DSS-induced colitis model. A schematic illustration of the curing schedule of the DSS-induced colitis study (a). The length of inflamed colon (b) and histopathological changes (c) were significantly improved by poly P in DSS-induced colitis mice. RT-PCR showed that the expression of mRNA of proinflammatory cytokines IL1*β* (d), TNF (e), and IL6 (f) was significantly decreased by poly P, while that of IFN-*γ* (g) was not. Immunohistochemical staining of CD61 showed the accumulation of platelets at the surface of the intestinal epithelia after treatment with poly P (h). An ELISA of plasma D-dimer levels showed no significant effect on the thrombotic condition by treatment with poly P (i). The error bars and numbers show the standard deviation. ^∗^*P* < 0.05 by a one-way ANOVA followed by Fisher's protected least post hoc test.

BALB/c mice were allowed free access to drinking water containing 2.5% DSS for 5 days. PBS or 5 *μ*g of poly P was orally administered to mice once per day from day 0 to day 7, after which the mice were euthanized (Figure 1(a)). The colon length of PBS-treated mice was significantly shortened by 2.5% DSS treatment compared to the control mice, while the length in 5 *μ*g poly P-treated mice significantly recovered (Figure 1(b)). Hematoxylin-eosin (H&E) staining of the colonic sections showed that the exacerbation of the histopathological inflammation score induced by DSS treatment was significantly improved following treatment with poly P (Figure 1(c)). RT-PCR showed that the augmentation of the proinflammatory cytokines IL-1*β*, TNF, and IL-6 was significantly lower in the DSS + poly P group than in the DSS + PBS group (Figures 1(d)–1(g)). Interestingly, the accumulation of platelets was detected at the surface of the mucosa with the exfoliation of epithelia on H&E staining and immunohistochemical staining of CD61, which is a known surface antigen of platelets. The CD61-positive area of the DSS + poly P group was significantly larger than that of the DSS + PBS group (Figure 1(h)), suggesting that poly P induced the accumulation of platelets in the surface of the damaged epithelium due to intestinal inflammation.

To assesses systemic thrombotic tendency, the plasma D-dimer levels in the DSS-induced model mice were measured. Although DSS treatment increased the D-dimer levels, there was no significant difference between the DSS + PBS group and DSS poly P group (Figure 1(i)).

### 3.2. Poly P Induced Platelet Aggregation

To assess whether or not poly P directly induced the aggregation of platelets, poly P was added to the PRP obtained from the collected blood samples, and a platelet aggregation assay was performed. The absorption at 405 nm in suspended platelets was significantly decreased by the treatment with poly P (ratio of OD_405nm_ at 1000 sec: 0.636 ± 0.0072) as well as ADP (ratio of OD_405nm_ at 1000 sec: 0.817 ± 0.0246), which stimulates the aggregation of platelets, suggesting that poly P directly induced platelet aggregation.

**Figure 2:**
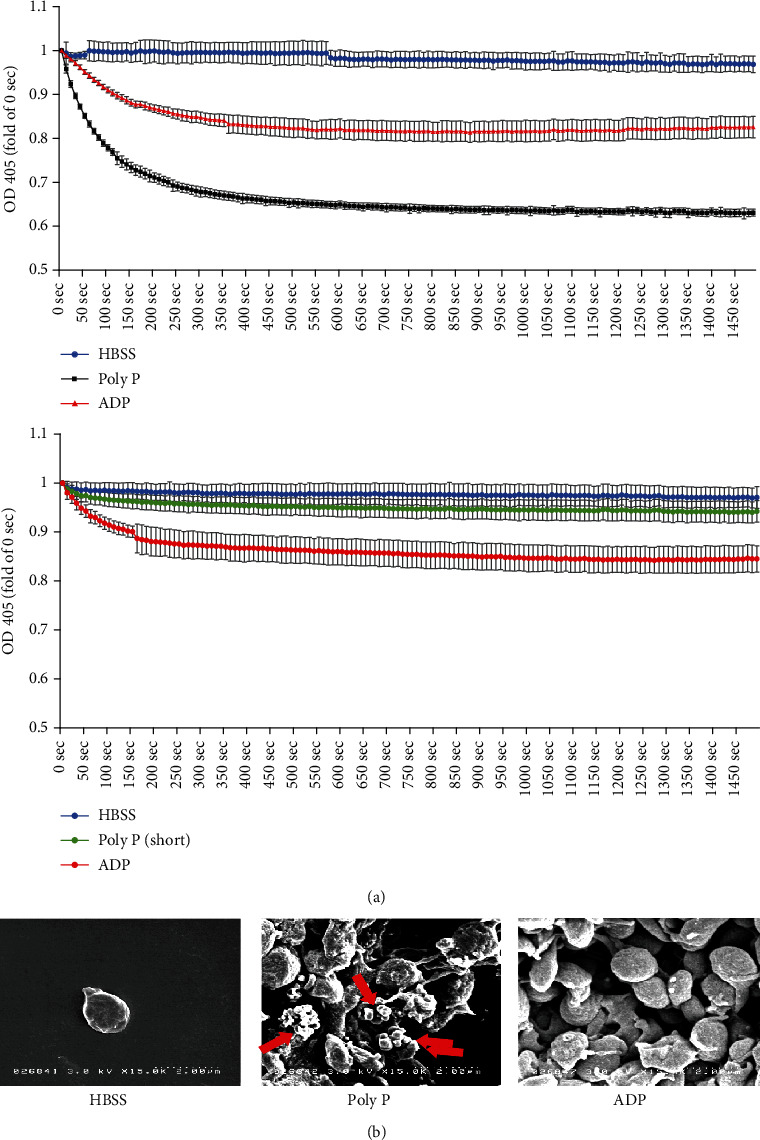
Poly P induced platelet aggregation. A platelet aggregation assay showed that long-chain poly P as well as short-chain poly P induced platelet aggregation and the effect of long-chain poly P on platelet aggregation was stronger than that of ADP (a). An SEM analysis indicated the morphological changes in naïve platelets by treatment with poly P or ADP (b). The arrow shows the insoluble particles, suspected of being poly P nanoparticles. The error bars and numbers show the standard deviation.

It was previously reported that short-chain polyphosphate, whose chain length is under 60 mer, endogenously exists in platelets and is associated with platelet aggregation [20–22]. Short-chain polyphosphate also induced the aggregation of platelets (ratio of OD_405nm_ at 1000 sec: 0.9445 ± 0.021), but the degree of the aggregation in the short-chain polyphosphate treatment group was lower than that in the poly P treatment group (Figure 2(a)).

To confirm the aggregation of platelets, an electron microscopic analysis was performed. The platelets aggregated following treatment with poly P as well as ADP and insoluble particles, which appeared to correspond to poly P nanoparticles, attached to the platelet surface (Figure 2(b)). These data suggest that poly P directly interacted with and activated the naïve platelets, resulting in the aggregation of platelets.

### 3.3. Poly P Improved the Wound Healing of Intestinal Epithelia Cells Mediating Platelet Activation

**Figure 3:**
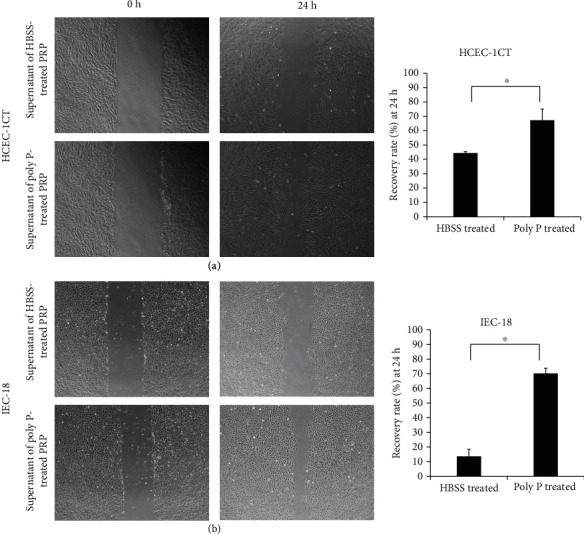
The supernatant of poly P-treated PRP increased the wound healing effect in epithelial cells. A cell scratch assay showed that the supernatant of poly P-treated PRP accelerated the closure of wound in HCEC-1CT (a) and IEC-18 (b) cells. The error bars and numbers show the standard deviation. ^∗^*P* < 0.05 by Student's *t*-test.

To assess whether or not poly P exerts wound healing effects through the activation of platelets, the supernatant of poly P-treated human PRP was collected and a cell scratch assay was performed. The supernatant of poly P-treated human PRP significantly improved the scratched wound compared to the supernatant of HBSS-treated human PRP in human normal epithelial HCEC-1CT cells (Figure 3(a)). Likewise, the wound healing effect was confirmed when rat normal epithelial cells (IEC-18 cells) were treated with supernatant of poly P-treated rat PRP (Figure 3(b)). In contrast, poly P did not directly improve the epithelial wound of HCEC-1CT cells (Supplementary Figure 1A) nor did the supernatant of 40 *μ*M ADP-treated PRP has any such effect (Supplementary Figure 1B).

**Figure 4:**
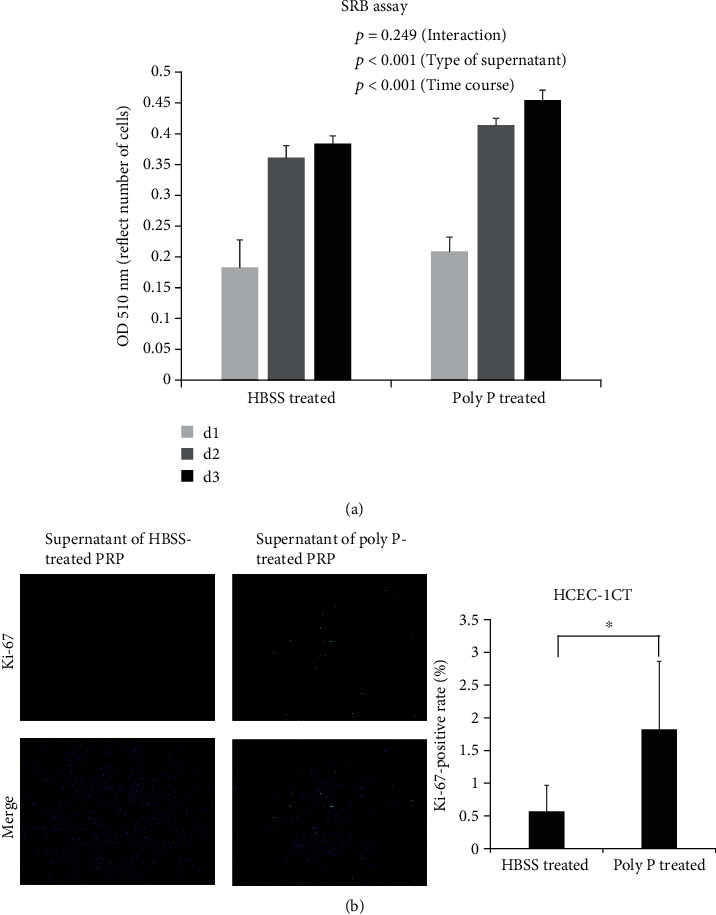
The supernatant of poly P-treated PRP promoted epithelial cell growth. An SRB assay showed that the supernatant of poly P-treated PRP significantly promoted cell growth (a) according to a two-factor repeated measure ANOVA analysis. Immunocytochemistry of ki-67 showed that the ki67-positive cells were significantly increased by treatment with the supernatant of poly P-treated PRP (b). The error bars and numbers show the standard deviation. ^∗^*P* < 0.05 by Student's *t*-test.

To assess whether or not a poly P-treated platelet-derived molecule promotes cell growth, an SRB assay was performed. The cell growth of HCEC-1CT cells was significantly increased following treatment with the supernatant of poly P-treated human PRP compared to treatment with the supernatant of HBSS-treated human PRP (Figure 4(a)). Immunocytochemistry showed that ki-67-positive cells were significantly more numerous following treatment with poly P-treated PRP (Figure 4(b)). These data suggest that poly P induced the secretion of growth-promoting molecules from platelets and thus promoted intestinal wound healing.

### 3.4. Poly P Induced the Release of Small Mediators from Platelets and Accelerated Cell Growth Mediating ERK Signaling

Previous investigations have shown that MAPK-associated molecules are closely associated with wound healing in numerous cell lines, including epithelial cells, keratinocyte, and cancer cells [23–28]. To clarify the growth-promoting mechanism of epithelial cells, the status of MAPK signaling was assessed.

**Figure 5:**
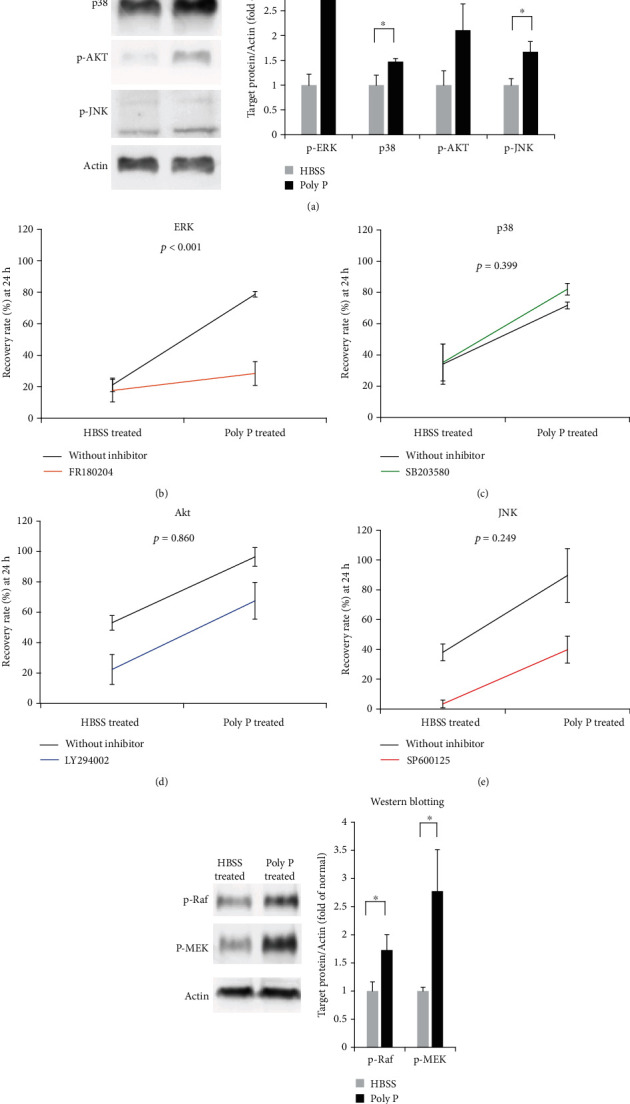
The supernatant of poly P-treated PRP activated the ERK signaling pathway and increased the wound healing effect in epithelial cells. Western blotting showed that the activation of ERK, p38, Akt, and JNK was induced by treatment with the supernatant of poly P-treated PRP (a). The activation of ERK, p38, Akt, and JNK was inhibited by FR180204, SB203580, LY294002, and SP600125, respectively. The wound healing effects by the supernatant of poly P-treated PRP were significantly decreased by the inhibition of ERK, but not p38, Akt, or JNK signaling (b–e) according to a two-factor ANOVA. Western blotting showed that upstream Raf-MEK-ERK signaling was activated (f). The error bars and numbers show the standard deviation. ^∗^*P* < 0.05 by Student's *t*-test (a, f).

Western blotting indicated that MAPK signaling-related molecules (ERK, p38, JNK, and AKT), especially those concerning the ERK signaling pathway, were significantly activated by the treatment of the supernatant of poly P-treated rat PRP in IEC-18 cells (Figure 5(a)). The epithelial healing effect of the supernatant of poly P-treated rat PRP was attenuated by treatment with an ERK inhibitor (FR180204) but not inhibitors of p38 (SB 203580), PI3K/Akt (LY294002), or JNK (SP600125) (Figures 5(b)–5(e)), suggesting that ERK signaling is responsible for promoting wound healing. Likewise, Western blotting also showed that the supernatant of poly P-treated rat PRP activated the upstream signaling of ERK, Raf-MEK signaling (Figure 5(f)), suggesting that poly P-induced molecules from platelets activated the epithelial Raf-MEK-ERK signaling pathway, resulting in the promotion of wound healing.

**Figure 6:**
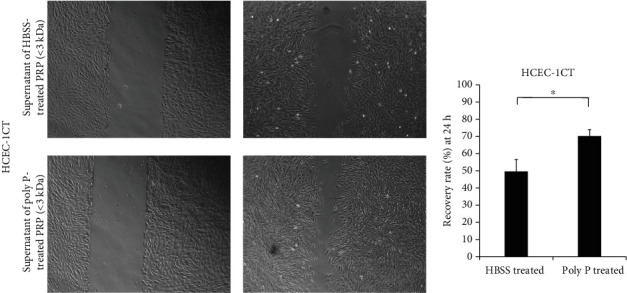
Poly P induced the expression of low-molecular weight mediators, which exerted wound healing effects, by platelets. The supernatant of poly P-treated PRP separated by the 3 kDa MWCO column exerted a wound healing effect. The error bars and numbers show the standard deviation. ^∗^*P* < 0.05 by Student's *t*-test.

Activated platelets are well known to release growth-related molecules to support wound healing [15, 29]. To identify the molecules released from platelets by poly P treatment, the growth factors in the supernatant of poly P-treated human PRP were assessed. An ELISA showed that the expression of PDGF, EGF, VEGF, and TGF-*β*, which are known to be components enclosing platelet granules [30–33], was not significantly increased in the supernatant of poly P-treated human PRP (Supplemental Figure 2). To clarify the molecular size of platelet-derived molecules, the supernatant of poly P-treated PRP was separated by the MWCO membrane. The wound healing effect was detected based on the flowthrough of a 3 kDa MWCO membrane (Figure 6), suggesting that low-molecular weight mediators supported intestinal wound healing.

## 4. Discussion

The present study showed that probiotic-derived poly P improved mucosal injury through inducing the accumulation of platelets at the inflamed mucosa in a DSS colitis mouse model. Poly P interacted with naïve platelets, promoted their aggregation without systemic thrombotic tendency, and induced the release of low-molecular weight molecules from platelets, resulting in epithelial wound healing of the colon. This report demonstrated for the first time that bacterial components can activate host platelets and induce the release of platelet-derived bioactive molecules, thereby exerting beneficial effects on their host.

Notably, the DSS colitis model analysis and platelet aggregation assay clearly showed that bacteria-derived poly P targeted not only host epithelia and immune cells but also host platelets. Our previous study demonstrated that poly P enhanced the intestinal barrier function through binding with integrin *β*1 of host epithelial cells [10]. Integrin *β*1 has been known to be located at the surface of platelets and play a pivotal role in the activation and granule secretion of platelets [34]. Our scanning electron microscope (SEM) analysis indicated that the granules, which were thought to be poly P particles, attached to platelets, suggesting that platelet integrin *β*1 signaling mediated the poly P effect of inducing the release of platelet-derived molecules, which exerted a wound healing effect on damaged tissue, including epithelial mucosa. The integrin *β*1 activator, including poly P, may be used to cure mucosal defects due to inflammatory disorders through the induction of platelets to the injured tissue.

Immunohistochemical staining of CD61 in DSS-poly P group mice showed that platelets accumulated at the surface of injured intestinal epithelium. A previous investigation showed that, in IBD patients with active inflammation, platelets moved with neutrophils from blood vessels to the intestinal lumen through the interstitial tissue [35, 36], indicating that the orally administered poly P activated host platelets at the surface of the injured intestinal epithelium and accelerated the process of epithelial wound healing.

A wound healing assay revealed that the supernatant of poly P-treated PRP, but not poly P itself, promoted the wound healing, indicating that platelet activation is indispensable for the poly P effect to be induced. In addition, ADP-treated PRP did not exert the same effect. A SEM analysis showed that the morphological change in platelet aggregation induced by poly P was quite different from that induced by ADP, suggesting that the mechanisms of platelet activation induced by poly P and ADP were not identical. We therefore searched for platelet-derived bioactive molecules induced by poly P. Platelets are known to contain myriad bioactive mediators in the *α*-granules and dense granules, which accelerate gastrointestinal wound healing. The most well-known molecules are growth factors, including platelet-derived growth factors (PDGF), epidermal growth factor (EGF), vascular endothelial growth factor (VEGF), and transforming growth factor-*β* (TGF-*β*). However, the present study showed that the releases of PDGF, EGF, VEGF, and TGF-*β* did not markedly differ following treatment with poly P-treated PRP. The acceleration of wound healing was also confirmed when using a 3 kDa MWCO-treated supernatant of poly P-treated PRP, indicating that poly P induced the release of small-sized mediators that were not considered growth factors because of their molecular size (more than 3 kDa). Further analyses are needed to identify the platelet-derived molecules mediating the wound healing effect of poly P.

MAPK and Akt signaling, including p38, JNK, ERK, and Akt, is well known to be involved in wound healing. Western blotting revealed that the wound healing effect of the supernatant of poly P-treated PRP was attenuated by the inhibition of the ERK signaling pathway but not the p38, JNK, or Akt pathways, whereas the activation of p38, JNK, and Akt as well as ERK was detected. The supernatant of poly P-treated rat PRP also activated Raf and MEK, which are upstream molecules of ERK, illustrating that the wound healing effect induced by the supernatant of poly P-treated PRP was mediated by the Raf-MEK-ERK signaling pathway. Our previous study revealed that poly P directly activated p38 MAPK in epithelial cells. These findings indicated that poly P promoted wound healing through the activation of the ERK signaling pathway by activating platelets and enhancing the intestinal barrier function through the direct activation of the p38 MAPK signaling pathway.

Platelets are considered to be inflammatory cells because proinflammatory molecules, including platelet-activating factor and macrophage inflammatory protein-1 *α*, are released by the activation of platelets, resulting in the exacerbation of inflammation [37–39]. In contrast, our results demonstrated that the molecules derived from poly P-activated platelets promoted epithelial wound closure without the enhancement of intestinal inflammation. Our previous studies indicated that poly P directly suppressed the cytokine release from the intestinal epithelia as well from macrophages [11], suggesting that the intestinal inflammation enhanced by platelet-derived proinflammatory molecules was cancelled by the direct anti-inflammatory effect of poly P. Otherwise, poly P may induce specific molecules that possesses a wound healing effect with no function in relation to the inflammation status. A further analysis to identify wound healing-associated molecules specifically derived from poly P-activated platelets is needed to elucidate the detailed mechanism of wound healing.

The risk of systematic thromboembolism in inflammatory bowel disease (IBD) is known to be high [40–44]. To apply poly P as a medicinal agent for IBD, we must determine the systematic thrombotic tendency, as light transmission aggregometry in the present study showed that poly P has a high platelet aggregation activity. In this regard, an ELISA showed that there were no significant differences in the plasma D-dimer level in DSS model mice between the DSS-PBS group and DSS-poly P group. Likewise, as shown in our previous report, thrombosis in UC patients was not observed in the first-in-human study of poly P. Furthermore, an assessment of the pharmacokinetics of polyphosphate in mice revealed that the daily oral administration of 10 mg polyphosphate did not increase the serum phosphate levels, and a rodent and nonrodent toxicity study revealed no evidence of thromboembolism [12]. Moreover, the immunohistochemical analysis showed that the CD 61 positivity was localized at the surface of the intestine. These findings suggest that orally administered poly P did not transit the gastrointestinal tract because of its high molecular weight and activated platelets located in the inflamed gastrointestinal mucosa.

## 5. Conclusions

We demonstrated that probiotic-derived poly P accelerated epithelial wound healing by enhancing ERK phosphorylation following host platelet activation, showing that poly P is a viable drug candidate for inducing mucosal healing in IBD. The activation of mammalian platelets by bacteria-derived molecules is a novel mechanism of host microbial interaction, which is a potential target for the treatment of gastrointestinal diseases.

## Data Availability

The data used to support the findings of this study are available from the corresponding author upon request.

## Supplementary Materials

Supplementary 1 Supplementary Figure 1. The poly P direct addition and supernatant of ADP-treated PRP did not promoted epithelial cell growth. A cell scratch assay showed that direct addition of final concentrations of 1, 10, and 100 *μ*g/mL Poly P did not improve the epithelial wound in HCEC-1CT cells (A). Supernatant of 40 *μ*M ADP-treated PRP also did not improve this wound (B).

Supplementary 2 Supplementary Figure 2. The components of platelet-derived growth factors did not change in the supernatant of poly P-treated PRP. An ELISA of PDGF, EGF, VEGF, and TGF-*β* showed that significant changes were not detected in the supernatant of poly P-treated PRP.

## References

[1] Nishida A., Inoue R., Inatomi O., Bamba S., Naito Y., Andoh A. (2018). Gut microbiota in the pathogenesis of inflammatory bowel disease. *Clinical Journal of Gastroenterology*.

[2] Gagnière J. (2016). Gut microbiota imbalance and colorectal cancer. *World Journal of Gastroenterology*.

[3] Wang M., Karlsson C., Olsson C. (2008). Reduced diversity in the early fecal microbiota of infants with atopic eczema. *The Journal of Allergy and Clinical Immunology*.

[4] Pace F., Pace M., Quartarone G. (2015). Probiotics in digestive diseases: focus on Lactobacillus GG. *Minerva Gastroenterologica e Dietologica*.

[5] Park M.-S., Song N.-E., Baik S.-H., Pae H.-O., Park S. H. (2017). Oral administration of lactobacilli isolated from Jeotgal, a salted fermented seafood, inhibits the development of 2,4-dinitrofluorobenzene-induced atopic dermatitis in mice. *Experimental and Therapeutic Medicine*.

[6] Matsuoka K., Uemura Y., Kanai T. (2018). Efficacy of Bifidobacterium breve fermented milk in maintaining remission of ulcerative colitis. *Digestive Diseases and Sciences*.

[7] Yan F., Cao H., Cover T. L., Whitehead R., Washington M. K., Polk D. B. (2007). Soluble proteins produced by probiotic bacteria regulate intestinal epithelial cell survival and growth. *Gastroenterology*.

[8] Fujiya M., Musch M. W., Nakagawa Y. (2007). The _Bacillus subtilis_ quorum-sensing molecule CSF contributes to intestinal homeostasis via OCTN2, a host cell membrane transporter. *Cell Host & Microbe*.

[9] Kelly C. J., Zheng L., Campbell E. L. (2015). Crosstalk between microbiota-derived short-chain fatty acids and intestinal epithelial HIF augments tissue barrier function. *Cell Host & Microbe*.

[10] Segawa S., Fujiya M., Konishi H. (2011). Probiotic-derived polyphosphate enhances the epithelial barrier function and maintains intestinal homeostasis through integrin-p 38 MAPK pathway. *PloS one*.

[11] Kashima S., Fujiya M., Konishi H. (2015). Polyphosphate, an active molecule derived from probiotic _Lactobacillus brevis_ , improves the fibrosis in murine colitis. *Translational Research : The Journal of Laboratory and Clinical Medicine*.

[12] Fujiya M., Ueno N., Kashima S. (2019). Long-chain polyphosphate is a potential agent for inducing mucosal healing of the colon in ulcerative colitis. *Clinical Pharmacology and Therapeutics*.

[13] Ramos G. P., Papadakis K. A. (2019). Mechanisms of disease: inflammatory bowel diseases. *Mayo Clinic Proceedings*.

[14] Yu L. C.-H. (2018). Microbiota dysbiosis and barrier dysfunction in inflammatory bowel disease and colorectal cancers: exploring a common ground hypothesis. *Journal of Biomedical Science*.

[15] Eisinger F., Patzelt J., Langer H. F. (2018). The platelet response to tissue injury. *Frontiers in Medicine*.

[16] Berg D. J., Davidson N., Kühn R. (1996). Enterocolitis and colon cancer in interleukin-10-deficient mice are associated with aberrant cytokine production and CD4(+) TH1-like responses. *The Journal of Clinical Investigation*.

[17] Schneider C. A., Rasband W. S., Eliceiri K. W. (2012). NIH Image to ImageJ: 25 years of image analysis. *Nature Methods*.

[18] Vichai V., Kirtikara K. (2006). Sulforhodamine B colorimetric assay for cytotoxicity screening. *Nature Protocols*.

[19] Wu M., Stockley P. G., Martin W. J. (2002). An improved Western blotting technique effectively reduces background. *Electrophoresis*.

[20] Jain S., Pitoc G. A., Holl E. K. (2012). Nucleic acid scavengers inhibit thrombosis without increasing bleeding. *Proceedings of the National Academy of Sciences of the United States of America*.

[21] Morrissey J. H. (2012). Polyphosphate: a link between platelets, coagulation and inflammation. *International Journal of Hematology*.

[22] Smith S. A., Mutch N. J., Baskar D., Rohloff P., Docampo R., Morrissey J. H. (2006). Polyphosphate modulates blood coagulation and fibrinolysis. *Proceedings of the National Academy of Sciences of the United States of America*.

[23] Aoki K., Kondo Y., Naoki H., Hiratsuka T., Itoh R. E., Matsuda M. (2017). Propagating wave of ERK activation orients collective cell migration. *Developmental Cell*.

[24] Harper E. G., Alvares S. M., Carter W. G. (2005). Wounding activates p38 map kinase and activation transcription factor 3 in leading keratinocytes. *Journal of Cell Science*.

[25] Shin I., Kim S., Song H., Kim H.-R. C., Moon A. (2005). H-Ras-specific activation of Rac-MKK3/6-p38 Pathway. *The Journal of Biological Chemistry*.

[26] Saika S., Okada Y., Miyamoto T. (2004). Role of p38 MAP kinase in regulation of cell migration and proliferation in healing corneal epithelium. *Investigative Ophthalmology & Visual Science*.

[27] Lee J., Jang H., Park S. (2019). Platelet-rich plasma activates AKT signaling to promote wound healing in a mouse model of radiation-induced skin injury. *Journal of Translational Medicine*.

[28] Squarize C. H., Castilho R. M., Bugge T. H., Gutkind J. S. (2010). Accelerated wound healing by mTOR activation in genetically defined mouse models. *PloS one*.

[29] Anitua E., Andia I., Ardanza B., Nurden P., Nurden A. (2017). Autologous platelets as a source of proteins for healing and tissue regeneration. *Thrombosis and Haemostasis*.

[30] Barrientos S., Stojadinovic O., Golinko M. S., Brem H., Tomic-Canic M. (2008). Perspective article: growth factors and cytokines in wound healing. *Wound Repair and Regeneration*.

[31] Schultz G., Rotatori D. S., Clark W. (1991). EGF and TGF-*α* in wound healing and repair. *Journal of Cellular Biochemistry*.

[32] Salgado R., Benoy I., Bogers J. (2001). Platelets and vascular endothelial growth factor (VEGF): a morphological and functional study. *Angiogenesis*.

[33] Grainger D. J., Wakefield L., Bethell H. W., Farndale R. W., Metcalfe J. C. (1995). Release and activation of platelet latent TGF-*β* in blood clots during dissolution with plasmin. *Nature Medicine*.

[34] Petzold T., Ruppert R., Pandey D. (2013). *β*1 integrin-mediated signals are required for platelet granule secretion and hemostasis in mouse. *Blood*.

[35] Stokes K. Y., Granger D. N. (2012). Platelets: a critical link between inflammation and microvascular dysfunction. *The Journal of Physiology*.

[36] Weissmüller T., Campbell E. L., Rosenberger P. (2008). PMNs facilitate translocation of platelets across human and mouse epithelium and together alter fluid homeostasis via epithelial cell-expressed ecto-NTPDases. *The Journal of Clinical Investigation*.

[37] Klinger M. H. (1997). Platelets and inflammation. *Anatomy and Embryology*.

[38] Thomas M. R., Storey R. F. (2017). The role of platelets in inflammation. *Thrombosis and Haemostasis*.

[39] Franco A. T., Corken A., Ware J. (2015). Platelets at the interface of thrombosis, inflammation, and cancer. *Blood*.

[40] Ando K., Fujiya M., Nomura Y. (2018). The incidence and risk factors of venous thromboembolism in Japanese inpatients with inflammatory bowel disease: a retrospective cohort study. *Intestinal Research*.

[41] Grip O., Svensson P. J., Lindgren S. (2009). Inflammatory bowel disease promotes venous thrombosis earlier in life. *Scandinavian Journal of Gastroenterology*.

[42] van Wersch J. W., Houben P., Rijken J. (1990). Platelet count, platelet function, coagulation activity and fibrinolysis in the acute phase of inflammatory bowel disease. *Clinical Chemistry and Laboratory Medicine*.

[43] Andoh A., Yoshida T., Yagi Y. (2006). Increased aggregation response of platelets in patients with inflammatory bowel disease. *Journal of Gastroenterology*.

[44] Ando K., Fujiya M., Nomura Y. (2019). The incidence and risk factors of venous thromboembolism in patients with inflammatory bowel disease: a prospective multicenter cohort study. *Digestion*.

