# Supplementary figures and images for: Probiotic-Derived Polyphosphate Accelerates Intestinal Epithelia Wound Healing through Inducing Platelet-Derived Mediators
Source: Mediators Inflamm. 2021 Mar 29;2021:5582943. doi: 10.1155/2021/5582943 (PMC8025129; doi:10.1155/2021/5582943)

Supplemental Figure 1

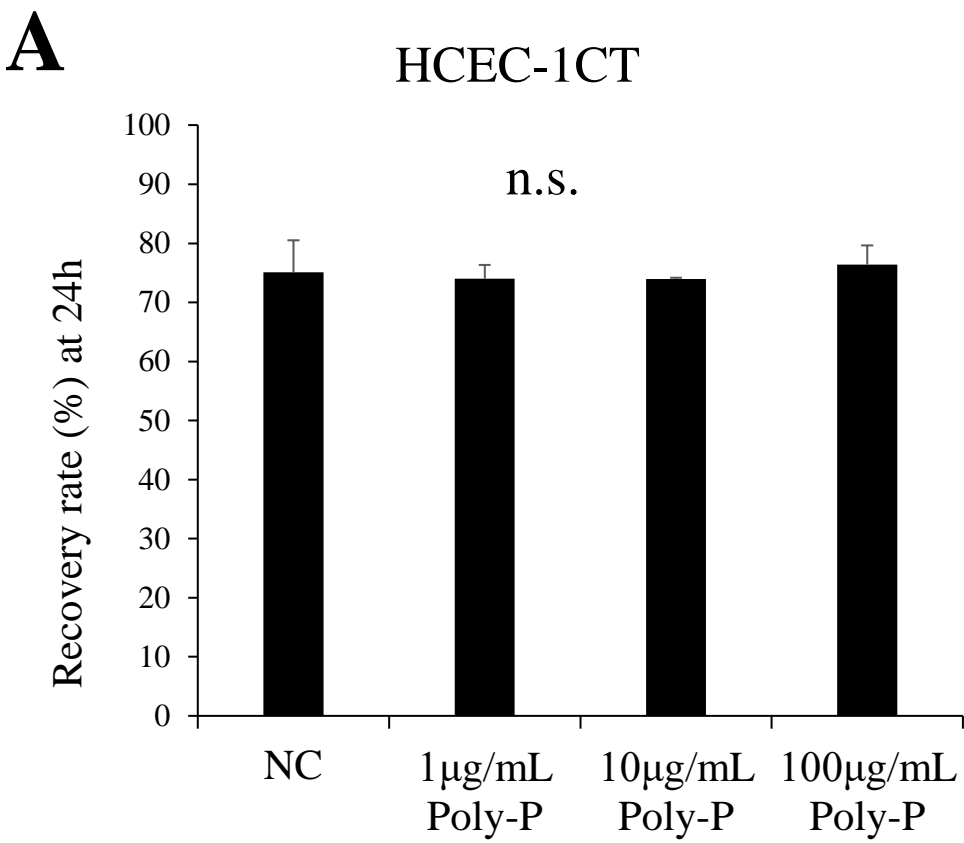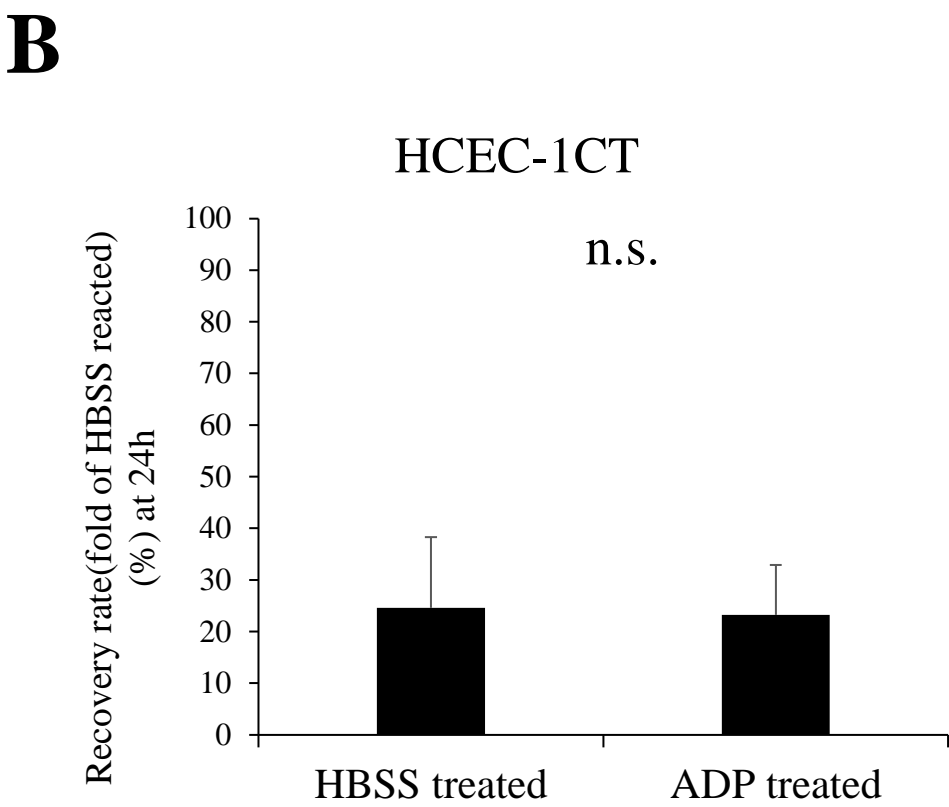

Supplement: Supplementary 1 — Supplementary Figure 1. The poly P direct addition and supernatant of ADP-treated PRP did not promoted epithelial cell growth. A cell scratch assay showed that direct addition of final concentrations of 1, 10, and 100 μg/mL Poly P did not improve the epithelial wound in HCEC-1CT cells (A). Supernatant of 40 μM ADP-treated PRP also did not improve this wound (B). [file 5582943.f1.pdf]

**Supplemental Figure 2**

n.s.

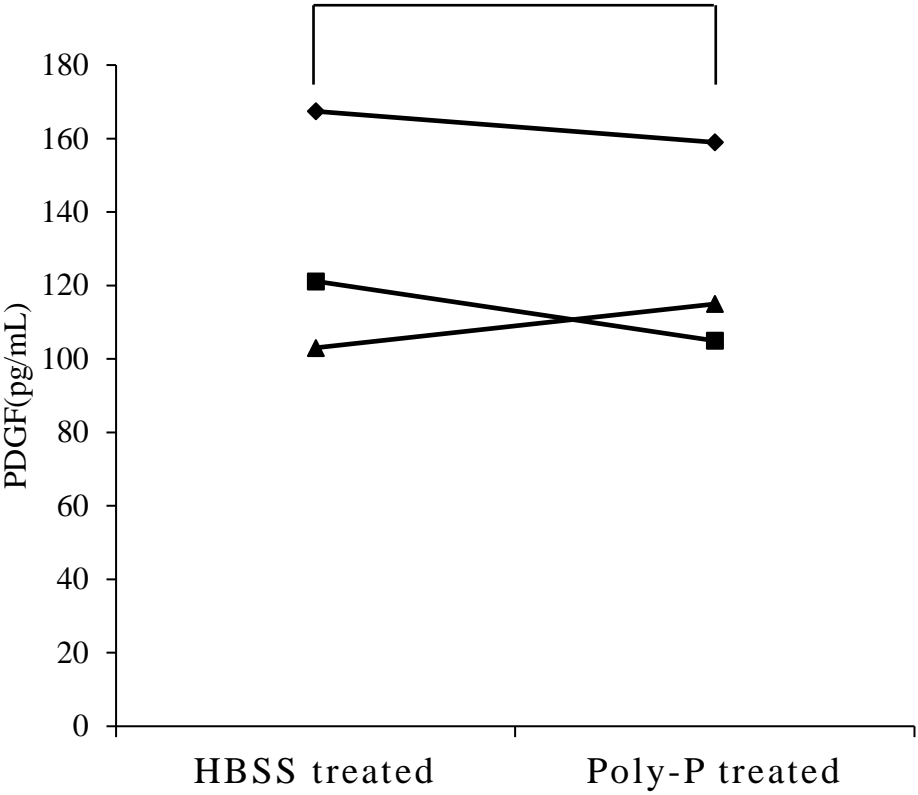

n.s.

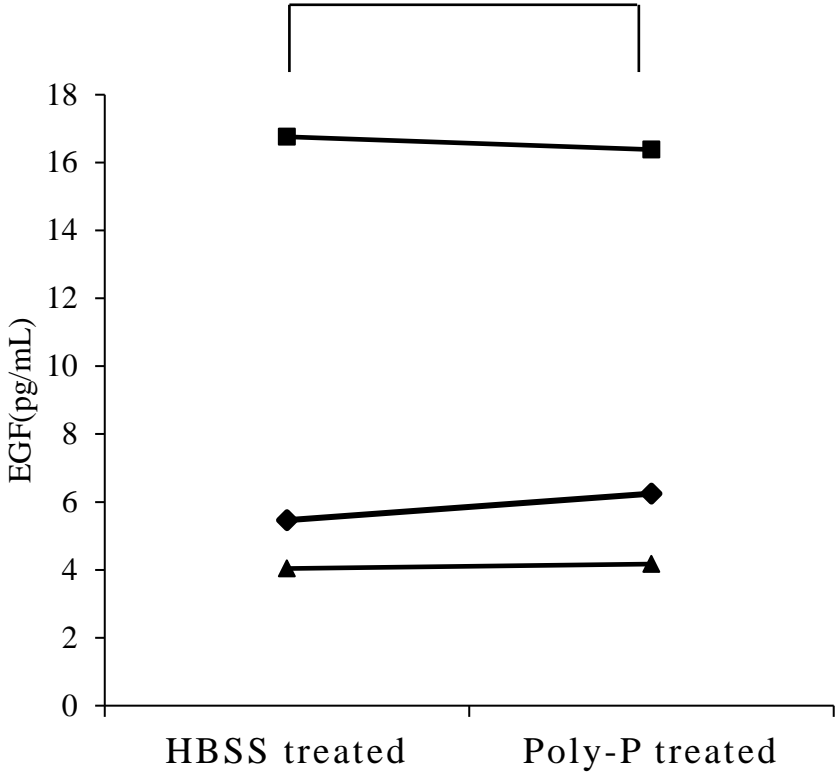

n.s.

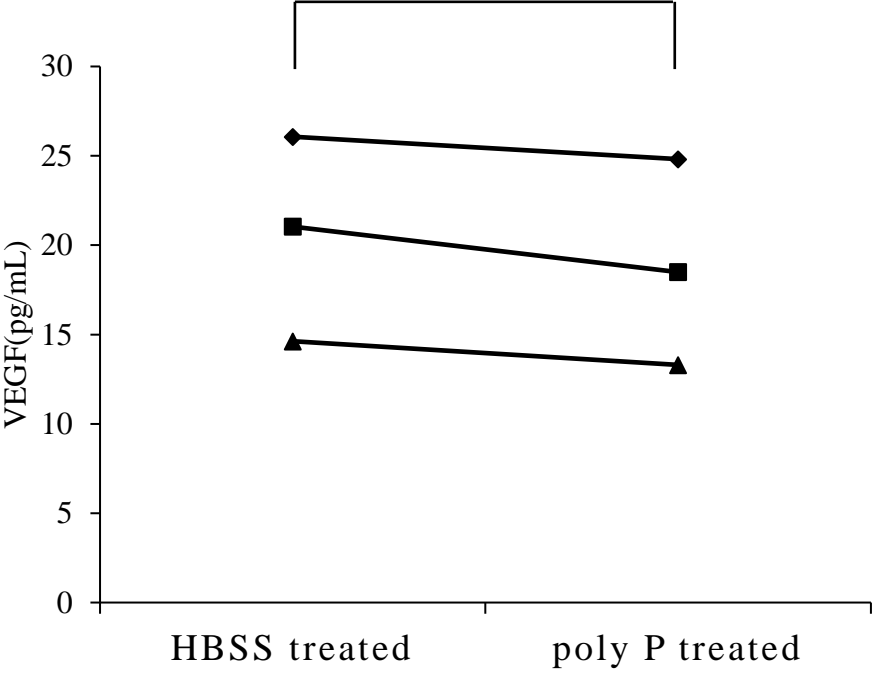

n.s.

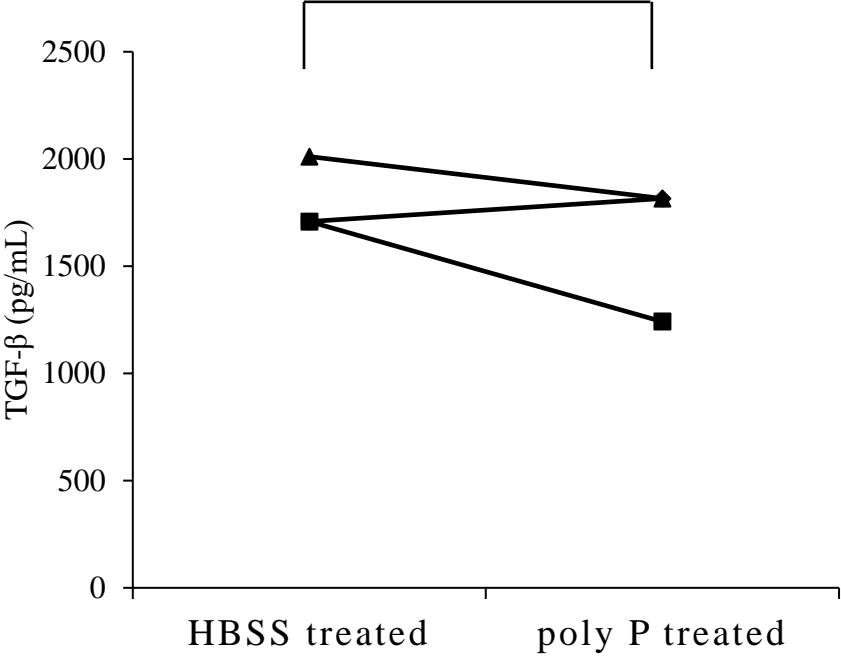

Supplement: Supplementary 2 — Supplementary Figure 2. The components of platelet-derived growth factors did not change in the supernatant of poly P-treated PRP. An ELISA of PDGF, EGF, VEGF, and TGF-β showed that significant changes were not detected in the supernatant of poly P-treated PRP. [file 5582943.f2.pdf]
